# Can I speak to the manager? The gender dynamics of decision-making in Kenyan maize plots

**DOI:** 10.1007/s10460-023-10484-w

**Published:** 2023-07-31

**Authors:** Rachel C Voss, Zachary M. Gitonga, Jason Donovan, Mariana Garcia-Medina, Pauline Muindi

**Affiliations:** 1 Sustainable Agri-food Systems Program, International Maize & Wheat Improvement Center (CIMMYT), ICRAF House, United Nations Avenue, Gigiri, Nairobi, Kenya; 2 Sustainable Agri-food Systems Program, International Maize & Wheat Improvement Center (CIMMYT), Carretera México-Veracruz, Km. 45, El Batán, Texcoco, 56237 Mexico; 3 Knowledge, Technology and Innovation Group, Wageningen University & Research, Droevendaalsesteeg 4, 6708 PB, Wageningen, Netherlands

**Keywords:** Gender, Maize, Decisions, Seed systems, Intrahousehold, Jointness

## Abstract

Gender and social inclusion efforts in agricultural development are focused on making uptake of agricultural technologies more equitable. Yet research looking at how gender relations influence technology uptake often assumes that men and women within a household make farm management decisions as individuals. Relatively little is understood about the dynamics of agricultural decision-making within dual-adult households where individuals’ management choices are likely influenced by others in the household. This study used vignettes to examine decision-making related to maize plot management in 698 dual-adult households in rural Kenya. The results indicated a high degree of joint management of maize plots (55%), although some management decisions—notably those related to purchased inputs—were slightly more likely to be controlled by men, while other decisions—including those related to hiring of labor and maize end uses—were more likely to be made by women. The prevalence of joint decision-making underscores the importance of ensuring that both men’s and women’s priorities and needs are reflected in design and marketing of interventions to support maize production, including those related to seed systems, farmer capacity building, and input delivery.

## Introduction

Gender responsiveness and gender intentionality in agricultural technology design and promotion have emerged as focal areas in agricultural development. This focus is fueled by evidence of unequal adoption of new technologies (e.g., seeds, fertilizer, and equipment) among men and women that is thought to contribute to a gender productivity gap (Doss 2001; Peterman et al. 2014). However, our understanding of the gender gap in uptake of new technologies is based on several assumptions about farm management. Primary among these is the idea that men and women are independent actors who choose, use, and benefit from technologies as individuals rather than as households or groups. This dichotomy is embodied in long-running discussions around “women’s crops” and “men’s crops,” although research suggests that gendered divisions of labor and management in smallholder systems are often much more ambiguous and mutable than these labels suggest (Doss 2002; Geisler 1993; Orr et al. 2016).

Common requirements for gender-disaggregated data in technology adoption research and impact evaluations are crucial for understanding women’s experiences, but these rely heavily on the assumption that men and women are independent actors. Male- and female-headed households are often compared as proxies for gender, for example, in studies of improved maize seed adoption (Adam et al. 2020a, b; Bezu et al. 2014; Kassa 2013; Kassie et al. 2017; Simtowe et al. 2019; Smale and Olwande 2014). This approach is problematic for multiple reasons, including the fact that definitions of household headship vary by context (Budlender 2003; Posel 2001; Rogan 2016) and that there are major differences in household demographic composition and resource access between male- and female-headed households (Doss 2015). Even more fundamentally, studies that use household headship as a proxy for gender typically neglect the circumstances of women in “male-headed households,” although these comprise, on average, half of rural women (Patel 2020).

Technology adoption in smallholder agriculture can also be broken down to the sub-household level and attributed to individuals according to plot management or ownership. While preferable in some ways to household-level analysis, this approach forces the reduction of dynamic farm management arrangements, including intrahousehold discussion and negotiation, into simple and discrete categories of individual management. For instance, most studies on maize seed uptake that do *not* compare female- and male-headed households assume a gender-based separation of maize plots to compare seed choice between individual men and women (Doss and Morris 2001; Marenya et al. 2021; O’Brien et al. 2016) or between men’s and women’s plots (Fisher and Carr 2015; Ndiritu et al. 2014; Teklewold et al. 2020). The pivot to individual-level analysis presents its own challenges and assumptions, including unclear definitions of what constitutes control over a plot. This can lead to intrahousehold disagreement and superficial understandings of intrahousehold farm management dynamics, especially around joint plot management and decision-making (Doss and Quisumbing 2020).

### Understanding plot management

Analyses of technology adoption at the plot level typically disaggregate based on plot management, ownership, or a general sense of plot “control.” This presents several challenges. First, individual interpretations of ownership and management and reports of technology uptake can vary greatly by respondents within households (Magnan et al. 2020; O’Brien et al. 2016; Slavchevska et al. 2021). A remarkable number of studies fail to specify who was interviewed for a household survey, although most rely on the household head to report on farm management for the household. Concerns about reliance on single respondents in households with multiple adults are not new (Fisher et al. 2010; Kilic et al. 2021; Magnan et al. 2020), and recent evidence highlights the prevalence of intrahousehold disagreement on questions ranging from assets to decision-making (Acosta et al. 2020; Ambler et al. 2021; Anderson et al. 2017; Seymour and Peterman 2018; van Campenhout et al. 2022).

In addition to challenges of intrahousehold disagreement are inconsistencies in the definitions of management that researchers and their respondents use. Plot management might refer to day-to-day maintenance of plots or to decision-making authority, although which decision(s) constitute management must be defined. In the literature, management sometimes goes undefined (e.g., Adam et al. 2020b; Ndiritu et al. 2014). In other cases, distinctions between decision-making, management, and control are unclear (de Brauw 2015). Slavchevska et al. (2021) examined household survey data from six African countries and found that the “plot manager” indicator, depending on the country and survey, referred variously to (1) those family members who made decisions about planting, (2) those who made decisions regarding planting and inputs, (3) to the person who “works the plot” and (4) the person who “manages” the plot. Many maize and fertilizer studies define management in reference to control over multiple activities or decisions (Burke and Jayne 2021; Chirwa 2005; Marenya et al. 2015; Teklewold et al. 2020). Among these are studies that rely on World Bank LSMS-ISA data, for which management is assigned according to who “makes primary decisions concerning crops to be planted, input use, and the timing of cropping activities.” The implicit assumption that the same individual controls all decisions or activities for a given plot with relative independence is not grounded in evidence.

### Understanding jointness

Beyond the ambiguous definitions of management lies the complex issue of “jointness.” Joint management is an important concept in intrahousehold adoption studies given that farm management decisions (e.g., which crops to grow, which inputs to use, and how to allocate labor) are inevitably influenced by the wider household context (Doss and Quisumbing 2020). Jointness in crop production has received increased attention in recent years (Acosta et al. 2020; Badstue et al. 2020; Doss 2018; Doss and Quisumbing 2020; Mohammed et al. 2022). However, interpretations vary; joint management can refer to either sharing or partitioning all or some responsibilities with varying degrees of participation and influence by the parties involved. One study of maize farmers in Uganda found that farmer perceptions of joint decision-making encompassed a range of decision-making structures and significant variation in women’s influence (Acosta et al. 2020). Similar findings emerged in dual-adult households in Ethiopia, where men reported consulting with their wives only to inform them about their decision (O’Brien et al. 2016). The degree of joint involvement may vary by crop and decision. For maize in Uganda, Shibata et al. (2020) reported a high prevalence of joint decision-making around how much to grow and what to do with profits from certain crops, but not all.

It is still largely unclear, in the context of technology adoption, how to handle joint management. Should both managers be counted as adopters or neither, and on what basis could joint adoption be ascribed to one individual? The literature on maize seed adoption suggests that many studies either do not encounter jointness (an unlikely reality) or do not accommodate it in data collection and analysis. Many studies use only a binary variable for gender of plot manager (Chirwa 2005; Fisher et al. 2019). World Bank LSMS-ISA surveys on which much gender analysis of productivity is based generally require the manager to be a single individual, which may push shared plots into men’s camp given their tendency to have the final say in many decisions. Other studies treat joint management as equivalent to sole management by men or women (e.g., Fisher and Carr 2015).

The exclusion of jointness from adoption research is problematic for several reasons, but mainly because joint production appears to be common. Ndiritu et al. (2014) found that 40% of plots studied in Kenya were jointly managed, making these the most common type of plot in the study. This prevalence is comparable to what Teklewold et al. (2020) found for maize plots in Uganda (38% joint) and Tanzania (47% joint), although in Malawi and Mozambique, joint plots appear less common (24% and 21% of plots, respectively) (Burke and Jayne 2021; Marenya et al. 2015). In most of these studies, joint plots are broken out and treated separately. This approach is logical but does not satisfy the broader need for gender-disaggregated data that clearly show technologies’ impacts on women. Ultimately, a better empirical understanding of joint management dynamics is needed to generate actionable guidance for delivery of new technologies to women and men in dual-adult households.

### Understanding household decision-making

As reviewed above, research on technology adoption and plot-level management has operated with a certain degree of muddiness concerning intrahousehold dynamics. The resulting assumption about men’s and women’s independent decision-making stands in stark contrast with gender research that emphasizes women’s limited autonomy and bargaining power within farming households (Badstue et al. 2020; Farnworth et al. 2020a, b; Petesch et al. 2017). The realities of women’s agency in intrahousehold decision-making around crop production remain a point of confusion linked to scholars’ differing research questions, approaches, and assumptions.

Recent studies on intrahousehold decision-making have provided important insights. Research from sub-Saharan Africa (Donald et al. 2017; van Campenhout et al. 2022) and Bangladesh (Ambler et al. 2021; Seymour and Peterman 2018) found that men and women perceived their involvement in farm management and decision-making in systematically different and often context-dependent ways. In Uganda, Shibata et al. (2020) interviewed one household head per household (alternating gender) on decision-making around crop end uses and income uses, finding men were more likely to report they made decisions independently, while women were more likely to report that decisions were joint or their spouse’s. Seymour and Peterman (2018) found a similar pattern in Bangladesh and Ghana across several household decisions. In Senegal, Bernard et al. (2020) documented a range of household decision-making scenarios and a diversity of rationales behind them. Together, these studies indicate that the intrahousehold dynamics of crop management are more complex than is typically assumed in either gender-disaggregated econometric analyses of technology adoption or many studies of women’s empowerment.

This study has two primary contributions. First, in a departure from existing research, we assessed the dynamics of crop management as a function of multiple production- and consumption-related decisions. In doing so, we unpacked implicit assumptions about what constitutes plot management and gained greater insight into joint management dynamics. Second, in response to contradictory claims from gender research and technology adoption research concerning women’s autonomy in farming decisions, we leveraged new methods to gain clarity around women’s involvement in crop management—not to be confused with women’s farm labor contributions, which have been more extensively studied. In pursuit of this, we drew on Bernard et al.’s (2020) approach to studying decision-making around livestock, using vignettes as a practical and less-invasive tool to assess household dynamics. Vignettes were used to understand a range of crop management decisions and their rationales in dual-adult maize-growing households in Kenya, with intentional comparison of spousal perceptions of decision-making dynamics. Through this, we explored the following questions: (1) What are the intrahousehold decision-making structures through which maize production occurs? (2) How do perceptions of and rationales behind these structures differ between spouses? and (3) How do crop management dynamics differ across a range of farming activities?

## Methods

### Study area and sampling strategy

This study targeted dual-adult, spousal-couple households in Kenya that grow maize, as joint contributions to and management of maize plots is known to be common (Adam et al. 2020a, b; Doss and Quisumbing 2020; Marenya et al. 2015; Ndiritu et al. 2014). The exclusion of single-adult households (or dual-adult households in which one spouse was absent for a prolonged period during data collection) was a necessary limitation of the study. Pretesting was carried out from October to November 2021 in the counties of Machakos (dry transitional altitude) and Kirinyaga (moist transitional altitudes, central Kenya), and data collection ultimately completed in December 2021 to February 2022 in Kirinyaga (moist transitional-altitude central Kenya) and Kakamega (higher-altitude western Kenya). Kirinyaga and Kakamega represent two important agroecological zones for maize production with differing farm structures and ethnic makeup.

We employed a two-stage probability proportional to size sampling technique with replacement, stratified by sub-counties. In the first stage, the number of sub-locations (clusters) targeted in each sub-county was determined according to the population weight of that sub-county in the county, for a total of 28 sub-locations in Kirinyaga and 30 in Kakamega. In the second stage, we sampled a consistent number of individuals per sub-location, ensuring that each individual in the county had the same probability of being included in the sample. Sample size calculations based on existing household data led to targeting of ten households in each of thirty sublocations in each county. To compensate for non-response and optimize field team resources, twelve households in each sub-location were randomly selected from a complete sampling list provided by local authorities, with additional randomly selected households used as replacement. The total sample was 698 households that cultivated 839 maize plots in total. In each household, men and women spouse co-heads (as identified by present household members) were invited to participate.

### Data collection

Prior to the launch of formal data collection, the research team conducted four gender-disaggregated focus group discussions with maize farmers and pretested survey tools in the research areas. These activities aided in refinement of the vignettes (described below) and the survey tool. Data were collected by a well-trained team of enumerators (three men and three women) who worked in mixed-gender pairs. All participants provided individual verbal consent and were modestly compensated for their time.

Data collection involved a two-part survey and lasted approximately an hour. A man and woman enumerator conducted the first half of the survey with both spouses to capture household structure and farm and non-farm income-generating activities. A participatory farm mapping exercise aided discussions of crops planted, agricultural practices, seed sourcing on maize plots, and end-uses of harvested maize in diverse maize plots. Data were collected at the plot level with a focus on the last long rainy season, and enumerators intentionally sought spousal agreement. As one objective of the study was to unpack the dynamics of plot management, respondents were *not* asked to identify the manager of each plot.

#### Vignettes to evaluate plot management structures

In the second half of the survey, spouses were separated for simultaneous individual interviews with enumerators of their gender. Discussions focused on how six maize management decisions were made for each maize plot. These decisions were selected based on past research with the objective of covering diverse aspects of management, including strategic decisions (choosing which maize variety to grow, how much fertilizer to use, and how to allocate the maize harvest), operational decisions (choice of planting date and how to allocate labor to production), and financial decisions (use of income derived from maize, in the case of sales).

Enumerators presented each spouse individually with a set of vignettes, or short stories, each of which depicted a household decision-making scenario. Spouses were asked to choose the vignette that they perceived to be best aligned with how each decision is made for each maize plot in their household. Vignettes have been used to understand agency and decision-making in previous studies (Bernard et al. 2020; Malapit et al. 2017) but have not been applied in reference to maize production in this context, or to such a range of management decisions. Vignettes can soften discussions of sensitive topics such as power dynamics, increase ease of response, reduce pressure on respondents to admit undesirable behaviors, and enable more participatory classification of responses (Bernard et al. 2020; Malapit et al. 2017; Martin 2006; Seymour and Peterman 2018). For this study, preliminary vignettes were developed using a combination of inductive and deductive methods, drawing on insights from Acosta et al.’s (2020) more general study of joint decision-making in households and Bernard et al.’s (2020) study of rationales behind decision-making structures around livestock. Once a set of draft vignettes was elaborated, gender-disaggregated focus group discussions were convened to review and refine them. Refined vignettes were then tested in the field to ensure context-appropriate wording, that the range of common decision-making structures were properly captured, ease of understanding and differentiating scenarios, amelioration of any gender sensitivity challenges, and adequate training of enumerators. Ultimately, refinement based on pretesting reduced the number of vignettes, removed strong language that seemed to affect respondents’ choices too heavily, and led to clearer distinctions between similar scenarios.

During the survey, enumerators read respondents the five vignettes and displayed flash cards to increase ease of retention and recall for the numerous scenarios. Vignettes offered to men and to women respondents are provided in Table 1 (*only* the narratives were read to research participants). Name placement and pronouns were swapped for men and women so that all participants heard vignettes phrased from the perspective of their gender. Collecting vignette data from both spouses enabled later assessment of intrahousehold accord and disagreement.

**Table 1:** Vignettes for the planting date decision

Vignette name (internal)	Interpretation	Women’s vignette narrative	Men’s vignette narrative
*Executive manager scenario*	Respondent is the primary decision-maker	Mary farms this plot, and she is the one who decides when to plant on this plot. She can listen to input from others, but it is ultimately her choice.	George farms this plot, and he is the one who decides when to plant on this plot. He can listen to input from others, but it is ultimately his choice.
*True jointness scenario*	Respondent and spouse collaborate on decision	Carol and Daniel farm together. They discuss when is the best time to plant on this plot and explain their reasoning. If Carol has good ideas about when to plant, Daniel listens to her. Sometimes they go with her choice, sometimes they go with his, and sometimes they compromise, but in the end, they decide together what is best.	Daniel and Carol farm together. They discuss when is the best time to plant on this plot and explain their reasoning. If Daniel has good ideas about when to plant, Carol listens to him. Sometimes they go with his choice, sometimes they go with hers, and sometimes they compromise, but in the end, they decide together what is best.
*Superficial jointness scenario*	Respondent participates in discussions but spouse has final say	Purity and Simon farm together, and they talk about when to plant on this plot. Purity sometimes disagrees with Simon and tells him what she thinks, but Simon ultimately decides when he thinks is best to plant.	Simon and Purity farm together, and they talk about when to plant on this plot. Simon sometimes disagrees with Purity and tells her what he thinks, but Purity ultimately decides when she thinks is best to plant.
*Marginalized spouse scenario*	Respondent is excluded from decision-making and is discontented with this exclusion	Lucy is interested in farming this plot, but her husband Jeffrey is more engaged with it. Lucy has good ideas about when to plant this plot, but Jeffrey decides on his own without her input. This is sometimes frustrating for Lucy.	Jeffrey is interested in farming this plot, but his wife Lucy is more engaged with it. Jeffrey has good ideas about when to plant this plot, but Lucy decides on her own without her input. This is sometimes frustrating for Jeffrey.
*Indifferent spouse scenario*	Respondent ‘opts out’ of decision-making and is content with this situation	Christina isn’t much engaged in farming this plot; her husband Isaac is the one who deals with the maize farming on this plot. Isaac chooses when to plant this plot. He and Christina don’t talk about this plot much, but this is okay because Christina trusts Isaac to make the best decision.	Isaac isn’t much engaged in farming this plot; his wife Christina is the one who deals with the maize farming on this plot. Christina chooses when to plant this plot. She and Isaac don’t talk about this plot much, but this is okay because Isaac trusts Christina to make the best decision.

After respondents identified the vignettes best aligned with their household decision-making model for each of the six management decisions, respondents were asked why each decision is made the way it is – in the spirit of Bernard et al. (2020) – and allowed to identify multiple rationales.

### Ethics approval

This study involved human subjects research and was performed in accordance with the ethical standards laid down in the 1964 Declaration of Helsinki. Ethics approvals were attained at the institutional level and Kenya country level via the International Livestock Research Institute (ILRI) Institutional Research Ethics Committee (#2021-61). COVID-19 precautions were adopted throughout. Verbal informed consent was obtained from all individual participants included in the study.

## Results

### Household maize production dynamics in Kenya

The first portion of the survey sought spousal agreement on household characteristic and farming practices, summarized in Table 2. On average, the reported farm size was 0.7 ha, slightly under half of which was dedicated to maize production. Most maize production was intended for household consumption; only one-third of households reported selling dry maize grain, and fewer sold fresh “green” maize. A large majority of households in both regions surveyed (81.7%) cultivated only a single maize plot and a small percentage cultivated two or three (no households cultivated more than three maize plots), leading to inclusion of 839 individual maize plots in the overall study. There was evidence that primary, secondary, and tertiary plots were systematically different in a few important ways—notably, secondary and tertiary maize plots were largely owned outside of the household (59.0% and 70.0%, respectively), meaning these were typically rented or borrowed. However, for analysis of decision-making dynamics at plot level, all household plots cultivated in the last rainy season were pooled.

**Table 2:** Sample household and farm characteristics, by county and in aggregate

		County			
		**Kakamega** (n = 365)	**Kirinyaga** (n = 333)	**Overall** (N = 698)	
**Demographics**	Age of the female co-head	46.23	49.06	47.58	
	Age of the male co-head	54.55	55.08	54.80	
	Household size	6.87	4.40	5.69	
**Land**	Total farm size (ha)	0.72	0.70	0.71	
	Maize area (ha)	0.36	0.32	0.34	
**Maize management practices ** (% of households)	Apply fertilizer (org. or inorg.)	96.70	99.10	97.85	
	Use pesticide	44.51	84.38	63.56	
	Irrigate maize	0.00	31.83	15.21	
**Primary source of labor ** (% of households)	Family labor	70.88	60.66	66.00	
	Community labor share	1.37	0.00	0.72	
	Hired labor	27.75	39.34	33.29	
**Maize production**	Yield (kg/ha)	2123	1265	1714	
**Maize sales ** (% of households)	Sold dry maize	32.05	36.34	34.10	
	Sold green maize	0.00	16.82	8.02	
	Reported no maize sales	67.95	46.84	57.88	
**Number of maize plots cultivated ** (% of households)	One	81.64	81.68	81.66	
	Two	16.71	17.12	16.91	
	Three	1.64	1.20	1.43	
**Number of maize varieties grown on the farm** (% of households)	One	89.32	94.59	91.83	
	Two	8.77	5.11	7.02	
	Three	1.37	0.30	0.86	

The vast majority of households (91.8% overall) reported growing only a single maize variety over the last long rainy season, and none reported more than three. Only 10% of households reported planting local varieties on their maize plots, although the accuracy of variety claims cannot be verified without genetic testing. For most plots, farmers acquired hybrids, improved open-pollinated varieties, or unknown varieties, typically from agrodealers but sometimes from NGOs or local shops (especially in Kakamega County, where One Acre Fund operates heavily). Couples were also asked to report who visited shops to acquire seed, revealing that 62.5% of hybrid seed purchases were made by the male household head and only 32.9% by the female household head. Respondents reported high use of inputs – 97.9% of households applied either organic fertilizer (67% of households) or inorganic fertilizer (91% of households), and 63.6% applied pesticides to their maize in the last season. For two-thirds of plots, the primary labor source was family labor.

### Decision-making structures around maize

The vignettes used to collect data on household decision-making structures (Table 1) were designed not only to capture joint decision-making and sole decision-making by the respondent, but also to capture nuance in scenarios in which the respondent was *not* the primary decision-maker. The popularity of individual vignettes (Table 3) thus provides useful insights, as does coding respondents’ vignette choices in terms of the gender of the primary decision-maker (Table 4). Table 3 includes instances in which decisions were made outside the household/couple or where they were not applicable – generally in cases where maize was never sold (making income-related decisions not applicable) or where input decisions were influenced by NGOs distributing materials.

**Table 3:** Vignette popularity varied by decision and respondent gender

Vignette selected (% of respondents)	Planting date decision			Variety decision			Fertilizer decision			Labor decision			Harvest use decision			Income use decision		
	Respondent		prtest	Respondent		prtest	Respondent		prtest	Respondent		prtest	Respondent		prtest	Respondent		prtest
	Women	Men	*p*-value	Women	Men	*p*-value	Women	Men	*p*-value	Women	Men	*p*-value	Women	Men	*p*-value	Women	Men	*p*-value
Executive manager	15.8	17.6	0.317	14.9	22.1	0.000*	11.6	29.1	0.000*	22.0	11.9	0.000*	18.4	4.4	0.000*	3.7	5.1	0.142
True jointness	70.5	68.5	0.384	66.8	62.6	0.068	65.5	56.7	0.000*	66.7	61.9	0.038*	74.0	66.0	0.000*	48.1	45.7	0.323
Superficial jointness	2.1	1.1	0.085	2.5	0.8	0.008*	1.4	0.0	0.001*	0.7	1.0	0.579	0.9	0.6	0.413	0.9	0.0	0.005*
Marginalized spouse	1.2	0.1	0.007*	1.5	0.1	0.001*	0.9	0.1	0.020*	0.8	0.1	0.035*	0.8	0.0	0.008*	0.8	0.1	0.035*
Indifferent spouse	9.7	11.7	0.184	12.8	13.4	0.715	16.9	9.7	0.000*	6.9	24.1	0.000*	4.4	28.4	0.000*	2.6	3.6	0.245
Outside the household	0.5	0.8	0.356	1.4	1.0	0.380	2.6	3.5	0.303	0.2	0.5	0.407	0.1	0.1	0.995	0.1	0.1	0.995
Not applicable	0.2	0.1	0.569	0.1	0.1	0.995	1.1	1.0	0.823	2.7	0.6	0.001*	1.3	0.5	0.072	43.8	45.4	0.506
Observations (plots)	847	839		847	839		847	839		847	839		847	839		847	839	

* p ≤ .05, indicating significant differences between men and women’s choice of each vignette for a management decision. Prtests assess the equality of proportions using large-sample statistics

**Table 4:** Gender of reported decision-maker inferred from vignette choice, disaggregated by decision and respondent. (Decisions made outside the household or not applicable have been removed.)

Gender of decision-maker (% of respondents)	Planting date decision			Variety decision			Fertilizer decision			Labor decision			Harvest use decision			Income use decision		
	Respondent		prtest	Respondent		prtest	Respondent		prtest	Respondent		prtest	Respondent		prtest	Respondent		prtest
	Women	Men	*p*-value	Women	Men	*p*-value	Women	Men	*p*-value	Women	Men	*p*-value	Women	Men	*p*-value	Women	Men	*p*-value
Man’s decision	16.2	17.9	0.322	15.2	22.7	0.000*	12.2	30.7	0.000*	22.7	12.2	0.000*	18.7	4.4	0.000*	6.7	9.5	0.104
Joint decision	70.8	69.1	0.364	67.9	63.1	0.068	67.8	59.1	0.000*	68.7	62.2	0.007*	75.2	66.3	0.000*	85.6	83.7	0.427
Woman’s decision	13.1	13.1	0.937	16.9	14.2	0.163	20.1	10.1	0.000*	8.6	25.6	0.000*	6.2	29.3	0.000*	7.7	6.7	0.555
Observations (plots)	845	838		846	838		838	831		824	834		835	836		476	458	

* p ≤ .05, indicating significant differences between men and women’s choice of decision-maker for each management decision

The “true jointness” vignette was the most commonly chosen scenario for all decision types, and was reported significantly more often by women than by men for three of the decisions (fertilizer use, labor allocation, and harvest use) (Table 3). The prevalence of joint decision-making varied slightly according to the decision; those most commonly made jointly concerned end uses for harvested maize and the use of income derived from maize. This finding is surprising given widely held assumptions about women’s limited involvement in financial decision-making (discussed later). Additionally, focus group discussions indicated that both men and women secretly engaged in small-scale, ad hoc maize sales to meet individual needs. Although this runs somewhat counter to the reports of joint decision-making in the survey, focus group participants described covert sales as fairly standard and generally acceptable.

Although joint decision-making was most common, the circumstances around sole decision-making are telling. First, and importantly, the results do not provide evidence of women’s or men’s widespread exclusion from decision-making around maize. As is clear in Table 3, the “superficial jointness” scenario, in which the respondent engaged in discussions and negotiations but did not have the final say, was very rarely selected (under 3% for all decisions). The “marginalized spouse” scenario, in which the respondent was entirely excluded from decision-making to their dissatisfaction, was even less common (under 2% for all decisions). Women in focus group discussions indicated the “marginalized spouse” scenario is rather old-fashioned and often reduces women’s interest in contributing labor to a plot. Instead, the “indifferent spouse” scenario, which presented the respondent as “opting out” of the decision-making process and contented with the arrangement, proved most popular by far among respondents who did not control decisions. This arrangement was particularly popular among men for labor allocation and harvest use decisions, indicating that many men were evidently comfortable leaving these choices to their wives. On the other hand, women were comfortable leaving the choice and purchase of fertilizer to their husbands.

### Spousal agreement and disagreement around decision-making structures

In aggregate, men and women’s reports of the gender of the decision-maker differed significantly for many management decisions (Table 4). Disagreement was particularly evident around fertilizer use, labor allocation, and harvest use decisions. While men tended to claim fertilizer decisions for themselves, women more often reported joint or woman-led decision-making. In contrast, women were significantly more likely to describe joint or men-led decisions around labor allocation and harvest use decisions, while more men assigned authority over these decisions to women.

**Fig. 1:**
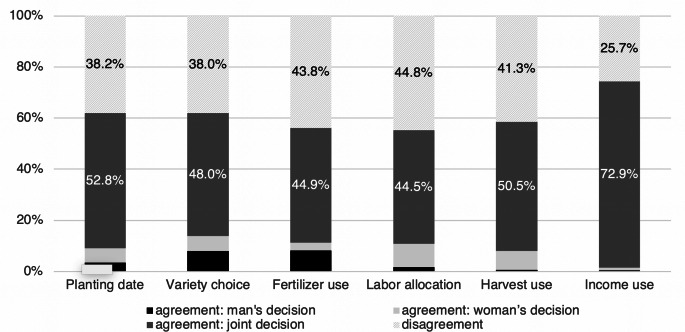
Spousal agreement (and disagreement) around gender of decision-maker across management decisions, at the household level

The reported gender of the decision-maker according to each spouse was used to assess agreement at the household level (Fig. 1). Spousal agreement was evident when the two respondents in a household selected vignettes that aligned in terms of who made a management decision. Disagreement was evident when the two respondents in each household selected contradictory vignettes for a single management decision (e.g., when one spouse reported making the decision themselves while the other reported making it jointly). Figure 1 shows a high degree of agreement about joint decision-making among men and women respondents, but also substantial rates of disagreement across the six management decisions. The data also suggest slightly greater agreement around women’s control over labor allocation and uses of harvested maize. Women appeared to have least exclusive control over decisions related to income uses (where jointness was, by far, the predominant model) and fertilizer use. Spouses were somewhat more likely to agree that men have exclusive control over decisions related to fertilizer use and variety selection, while men had less control over the generally joint decisions related to harvest uses and income uses.

Striking in these data was the level of disagreement among spouses on the decision-making structures in place. There was most agreement around use of income derived from maize (with 72.9% of households agreeing on joint decision-making). However, for two decisions (labor allocation and fertilizer allocation), disagreement between spouses occurred in nearly half of all households. This was not an unexpected result given existing research on spousal disagreement (Acosta et al. 2020; Ambler et al. 2021; Anderson et al. 2017; Seymour and Peterman 2018; van Campenhout et al. 2022).

Categorizing disagreement in meaningful ways was helpful in interpreting patterns. One category could be termed “full disagreement,” in which each spouse claimed sole control over a decision or each spouse assigned sole control to the other. In only a small proportion of households (under 10%, depending on the decision) was there “full disagreement.” Much more common were instances where one spouse reported sole decision-making and the other reported joint decision-making. To interpret these subtler disagreements, we built upon a framework proposed by Annan et al. (2020), which categorized the nature of disagreements according to whether they constituted women “taking power” by claiming more involvement in decisions than their husband reported for them, or men “giving power” by assigning their spouse more involvement in decisions than women reported themselves. We expanded this framework to consider different permutations of disagreement (Table 5).

**Table 5:** Spousal disagreement over who makes each management decision was categorized according to who “gives” authority and who “takes” authority. Spousal agreement was categorized according to the agreed-upon decision-maker

Decision-maker	Characterization	Rationale
*Spousal disagreement scenarios*		
**M respondent**: man decides **F respondent**: woman decides	Full disagreement/ both claiming authority (FDC)	Both respondents claim control over the decision. Neither cedes authority to the other.
**M respondent**: woman decides **F respondent**: man decides	Full disagreement/ both delegating authority (FDD)	Spouses delegate full decision-making authority to the other. Both cede authority over decision making.
**M respondent**: man decides **F respondent**: joint decision	Man taking power (MTP)	The man claims full control over decision-making and allocates himself more power than his spouse allocates him. The woman says it is a joint decision.
**M respondent**: joint decision **F respondent**: woman decides	Woman taking power (WTP)	The woman claims full control over decision-making and allocates herself more power than her spouse allocates her. The man says it is a joint decision.
**M respondent**: joint decision **F respondent**: man decides	Woman giving power (WGP)	The woman allocates her spouse full control over decision-making although he says it is a joint decision. The woman cedes power to her spouse.
**M respondent**: woman decides **F respondent**: joint decision	Man giving power (MGP)	The man allocates his spouse full control over decision-making although she says it is a joint decision. The man cedes power to his spouse.
*Spousal agreement scenarios*		
**M respondent**: man decides **F respondent**: man decides	Full spousal agreement	Both agree on decision-making by the man.
**M respondent**: joint decision **F respondent**: joint decision	Full spousal agreement	Both agree on joint decision-making.
**M respondent**: woman decides **F respondent**: woman decides	Full spousal agreement	Both agree on decision-making by the woman.

Using these categories of disagreement, we assessed the frequencies of different forms of disagreement across management decisions. “Other disagreements” refer to cases where, e.g., one spouse reported the decision is made outside the household. Table 6 supports the finding that, even considering spousal disagreement, control over input use decisions skewed slightly toward men and control over decisions related to labor allocation and uses of maize harvests skewed slightly toward women.

**Table 6:** Categorizing spousal disagreement for different management decisions showed that some skew slightly toward men’s control and others toward women’s

	Decision skews toward man			Decision skews toward woman			Full disagreement		Obs.
*Activity*	*% MTP*	*% WGP*		*% WTP*	*% MGP*		*% FDC*	*% FDD*	*N*
Planting date	31.1	21.7		21.7	18.5		5.5	1.6	309
Variety choice	33.6	19.9		20.2	19.9		4.6	2.0	307
Fertilizer use	41.8	22.3		12.5	14.0		7.7	1.8	337
Labor allocation	20.3	12.2		28.3	33.6		3.1	2.5	354
Harvest use	8.4	10.8		26.7	51.8		0.6	1.8	334
Income use	32.4	18.1		25.7	22.9		1.0	0.0	105

Abbreviations refer to characterizations in Table 5: MTP = man taking power, WGP = woman giving power, WTP = woman taking power, MGP = man giving power, FDC = full disagreement (claiming authority), FDD = full disagreement (delegating authority)

### Rationales behind decision-making structures

Rationales that individual respondents provided for joint decision-making structures (Table 7) suggested important gender-based differences in spouses’ motivations. Open-ended questions were asked and multiple rationales were allowed for each decision-making structure. While Table 7 reflects the frequencies of rationales averaged *across* the six management decisions studied, the specific rationales provided for jointness in each management decision are broken down in Table 8.

**Table 7:** Rationales provided for decision-making arrangements highlighted gender-based differences in priorities. Multiple rationales were allowed for each decision structure and thus do not total to 100%

Decision scenario	Reason	Men %	Women %	*p*-value
*Rationales provided for joint decision-making*	It is important to decide together to have the best outcome	68.3	78.5	0.000*
	It is important to decide together to avoid conflict or blame	54.8	19.2	0.000*
	It is important to decide together because the whole family benefits from the plot	54.4	46.2	0.000*
	We must agree because we both contribute labor and resources to the plot	46.9	52.6	0.000*
	We both have important knowledge to contribute to the decision	54.7	72.3	0.000*
	* Other(specify)	0.2	7.2	0.000*
*Rationales provided for claiming authority*	I am the only adult in the household when the decision is made	6.9	4.5	0.000*
	As head of household, I make this decision	11.9	12.5	0.078
	In our society, it is my responsibility to make this decision	5.5	15.6	0.000*
	Because I own the land, I make the decision	2.0	1.5	0.000*
	Because I work most on this plot, I make the decision	42.3	67.9	0.000*
	Because I have the money for this, I make the decision	37.6	21.0	0.000*
	I am more knowledgeable or skilled to make the decision	92.3	82.1	0.000*
	I make decisions about this activity/plot while my spouse decides for other things	27.9	40.8	0.000*
	Other(specify)	1.4	12.3	0.000*
*Rationales provided for ceding authority (‘indifferent spouse’ vignette)*	Other household head is the only adult in the household when the decision is made	18.8	4.05	0.000*
	To avoid conflict within the household, I let the other household head make this	21.0	25.0	0.000*
	As head of household, other household head makes this decision	0.6	21.7	0.000*
	In our society, it is the other household head’s responsibility to make this decision	17.2	2.8	0.000*
	Because other household head owns the land, they make the decision	0.0	5.8	0.000*
	Because other household head works most on this plot, they make the decision	37.0	21.5	0.000*
	Because other household head has the money for this, they make the decision	6.2	36.0	0.000*
	Other household head is more knowledgeable or skilled to make the decision	90.9	65.3	0.000*
	Other household head makes decisions about this activity/plot while I decide for other things	56.2	45.2	0.000*
	Other(specify)	2.8	13.6	0.000*

* p ≤ .05, indicating significant differences in the frequency with which men and women offered each rationale

**Table 8:** Rationales respondents provided for sharing authority (“true jointness” vignette) for each of the six decisions

Decision	Reason	Men %	Women %
Planting date	It is important to decide together to have the best outcome	62.6	80.4
	It is important to decide together to avoid conflict or blame	59.7	21.4
	It is important to decide together because the whole family benefits from the plot	47.5	41.5
	We must agree because we both contribute labor and resources to the plot	46.3	59.3
	We both have important knowledge to contribute to the decision	49.2	65.3
	Other(specify)	0.7	0.5
	*Observations*	575	597
Variety choice	It is important to decide together to have the best outcome	72.2	78.8
	It is important to decide together to avoid conflict or blame	53.9	18.9
	It is important to decide together because the whole family benefits from the plot	52.8	46.8
	We must agree because we both contribute labor and resources to the plot	41.3	42.9
	We both have important knowledge to contribute to the decision	64.57	78.3
	Other(specify)	0.4	0.0
	*Observations*	525	566
Fertilizer use	It is important to decide together to have the best outcome	72.1	76.2
	It is important to decide together to avoid conflict or blame	45.8	16.9
	It is important to decide together because the whole family benefits from the plot	58.0	32.4
	We must agree because we both contribute labor and resources to the plot	50.8	49.4
	We both have important knowledge to contribute to the decision	0.0	78.4
	Other(specify)	0.0	0.2
	*Observations*	476	555
Labor allocation	It is important to decide together to have the best outcome	64.2	74.3
	It is important to decide together to avoid conflict or blame	50.1	12.4
	It is important to decide together because the whole family benefits from the plot	47.6	46.2
	We must agree because we both contribute labor and resources to the plot	66.7	74.3
	We both have important knowledge to contribute to the decision	60.9	66
	Other(specify)	0.0	6.9
	*Observations*	519	565
Harvest use	It is important to decide together to have the best outcome	72.7	86
	It is important to decide together to avoid conflict or blame	67	24.6
	It is important to decide together because the whole family benefits from the plot	67.9	78.8
	We must agree because we both contribute labor and resources to the plot	26.9	22.3
	We both have important knowledge to contribute to the decision	54.0	69.7
	Other(specify)	0.4	4.3
	*Observations*	554	627
Income use	It is important to decide together to have the best outcome	73.63	87.7
	It is important to decide together to avoid conflict or blame	65.27	29.2
	It is important to decide together because the whole family benefits from the plot	71.54	71.5
	We must agree because we both contribute labor and resources to the plot	22.72	27.8
	We both have important knowledge to contribute to the decision	54.83	75.7
	Other(specify)	0.0	5.2
	*Observations*	333	407

In aggregate, the most common reason that both men and women gave for joint decision-making was that deciding together leads to the best outcomes. However, 35.6% more men than women cited the need to avoid conflict or blame as a reason for joint decision-making. Meanwhile, 17.6% more women than men responded that both spouses have important knowledge to contribute to the decision. This implies that women and men may have participated in joint decision-making with different motives–men in greater part to avoid intrahousehold conflict, and women in the belief that they could contribute important knowledge.

### Rationales behind women’s control of decisions

The rationales provided for women’s authority over decisions were captured in two places: in *women’s* reasons for identifying with the “executive manager” scenario and claiming authority, and in *men’s* reasons for identifying with the “indifferent spouse” scenario and ceding authority. Since “superficial jointness” and “marginalized spouse” scenarios were very seldom chosen, these were left out of this stage of analysis. Table 9 breaks down rationales for claiming authority and Table 10 breaks down rationales for ceding authority for each of the six management decisions.

**Table 9:** Rationales respondents provided for claiming decision-making authority for each of the six decisions

Decision	Reason	Men %	Women %
Planting date	I am the only adult in the household when the decision is made	9.5	8.2
	As head of household, I make this decision	16.2	14.9
	In our society, it is my responsibility to make this decision	10.8	4.5
	Because I own the land, I make the decision	4.1	2.2
	Because I work most on this plot, I make the decision	45.9	85.1
	Because I have the money for this, I make the decision	29.1	14.9
	I am more knowledgeable or skilled to make the decision	87.8	73.1
	I make decisions about this plot while my spouse decides for other things	25.7	46.3
	Other(specify)	0.0	2.2
	*Observations*	148	134
Variety choice	I am the only adult in the household when the decision is made	9.7	6.3
	As head of household, I make this decision	13.0	15.1
	In our society, it is my responsibility to make this decision	4.3	3.2
	Because I own the land, I make the decision	1.1	0.0
	Because I work most on this plot, I make the decision	45.4	69.8
	Because I have the money for this, I make the decision	35.1	40.5
	I am more knowledgeable or skilled to make the decision	96.2	83.3
	I make decisions about this activity/plot while my spouse decides for other things	33.5	37.3
	Other(specify)	0.0	3.2
	*Observations*	185	126
Fertilizer use	I am the only adult in the household when the decision is made	4.5	3.1
	As head of household, I make this decision	5.3	15.3
	In our society, it is my responsibility to make this decision	2.5	1.0
	Because I own the land, I make the decision	1.2	2.0
	Because I work most on this plot, I make the decision	39.3	77.6
	Because I have the money for this, I make the decision	48.8	49.0
	I am more knowledgeable or skilled to make the decision	94.3	76.5
	I make decisions about this activity/plot while my spouse decides for other things	27	37.8
	Other(specify)	0.4	4.1
	*Observations*	244	98
Labor allocation	I am the only adult in the household when the decision is made	4.0	3.2
	As head of household, I make this decision	10.0	8.6
	In our society, it is my responsibility to make this decision	4.0	10.8
	Because I own the land, I make the decision	2.0	2.2
	Because I work most on this plot, I make the decision	48.0	73.7
	Because I have the money for this, I make the decision	43.0	20.4
	I am more knowledgeable or skilled to make the decision	91.0	79.0
	I make decisions about this activity/plot while my spouse decides for other things	29.0	41.9
	Other(specify)	1.0	2.2
	*Observations*	100	186
Harvest use	I am the only adult in the household when the decision is made	13.5	3.8
	As head of household, I make this decision	24.3	13.5
	In our society, it is my responsibility to make this decision	10.8	43.6
	Because I own the land, I make the decision	2.7	1.3
	Because I work most on this plot, I make the decision	27.0	45.5
	Because I have the money for this, I make the decision	10.8	7.7
	I am more knowledgeable or skilled to make the decision	81.1	92.9
	I make decisions about this activity/plot while my spouse decides for other things	21.6	42.9
	I make this decision to prevent extra giving by the spouse	10.8	0.0
	Other(specify)	0.0	8.3
	*Observations*	37	156
Income use	I am the only adult in the household when the decision is made	7.0	3.2
	As head of household, I make this decision	39.5	6.5
	In our society, it is my responsibility to make this decision	16.3	0.0
	Because I own the land, I make the decision	4.7	0.0
	Because I work most on this plot, I make the decision	30.2	71.0
	Because I have the money for this, I make the decision	2.3	29.0
	I am more knowledgeable or skilled to make the decision	86.0	87.1
	I make decisions about this activity/plot while my spouse decides for other things	18.6	16.1
	Other(specify)	2.3	25.8
	*Observations*	43	31

**Table 10:** Rationales respondents provided for ceding decision-making authority (“indifferent spouse” vignette) for each of the six decisions

Decision	Reason	Men %	Women %
Planting date	Other household head is the only adult in the household when the decision is made	25.9	6.4
	To avoid conflict within the household	11.1	38.2
	As head of household, other household head makes this decision	0.0	23.6
	society norms	1.9	5.5
	Because other household head owns the land	0.0	9.1
	Because other household head works most on this plot	50.9	32.7
	Because other household head has the money for this, they make the decision	7.4	28.2
	Other household head is more knowledgeable or skilled to make the decision	83.3	60.0
	Other household head makes decisions about this activity/plot while I decide for other things	67.6	42.7
	Other (specify)	0.0	0.0
	*Observations*	108	110
Variety choice	Other household head is the only adult in the household when the decision is made	23.3	3.5
	To avoid conflict within the household, I let the other household head make this	22.5	23.2
	As head of household, other household head makes this decision	0.0	21.8
	In our society, it is the other household head’s responsibility to make this decision	3.3	1.4
	Because other household head owns the land, they make the decision	0.0	4.2
	Because other household head works most on this plot, they make the decision	53.3	15.5
	Because other household head has the money for this, they make the decision	11.7	45.1
	Other household head is more knowledgeable or skilled to make the decision	89.2	70.4
	Other household head makes decisions about this activity/plot while I decide for other things	54.2	43.0
	Other (specify)	0.8	0.0
	*Observations*	120	142
Fertilizer use	Other household head is the only adult in the household when the decision is made	17.1	2.5
	To avoid conflict within the household, I let the other household head make this	9.8	18.4
	As head of household, other household head makes this decision	1.2	16.0
	In our society, it is the other household head’s responsibility to make this decision	2.4	1.8
	Because other household head owns the land, they make the decision	0.0	2.5
	Because other household head works most on this plot, they make the decision	65.9	14.1
	Because other household head has the money for this, they make the decision	9.8	45.4
	Other household head is more knowledgeable or skilled to make the decision	89.0	77.3
	Other household head makes decisions about this activity/plot while I decide for other things	69.5	53.4
	Other (specify)	0.0	0.0
	*Observations*	82	163
Labor allocation	Other household head is the only adult in the household when the decision is made	19.9	8.6
	To avoid conflict within the household, I let the other household head make this	14.2	12.1
	As head of household, other household head makes this decision	0.0	6.9
	In our society, it is the other household head’s responsibility to make this decision	7.1	0.0
	Because other household head owns the land, they make the decision	0.0	5.2
	Because other household head works most on this plot, they make the decision	46.4	44.8
	Because other household head has the money for this, they make the decision	6.6	25.9
	Other household head is more knowledgeable or skilled to make the decision	87.7	63.8
	Other household head makes decisions about this activity/plot while I decide for other things	60.7	56.9
	Other (specify)	0.0	6.9
	*Observations*	211	58
Harvest use	Other household head is the only adult in the household when the decision is made	18.5	0.0
	To avoid conflict within the household, I let the other household head make this	29.0	27.0
	As head of household, other household head makes this decision	1.3	43.2
	In our society, it is the other household head’s responsibility to make this decision	0.0	10.8
	Because other household head owns the land, they make the decision	0.0	8.1
	Because other household head works most on this plot, they make the decision	18.9	29.7
	Because other household head has the money for this, they make the decision	3.4	8.1
	Other household head is more knowledgeable or skilled to make the decision	97.1	70.3
	Other household head makes decisions about this activity/plot while I decide for other things	49.6	35.1
	Other (specify)	0.0	18.9
	*Observations*	238	37
Income use	Other household head is the only adult in the household when the decision is made	3.3	13.6
	To avoid conflict within the household, I let the other household head make this	50.0	22.7
	As head of household, other household head makes this decision	0.0	54.5
	In our society, it is the other household head’s responsibility to make this decision	0.0	9.1
	Because other household head owns the land, they make the decision	0.0	9.1
	Because other household head works most on this plot, they make the decision	20.0	18.2
	Because other household head has the money for this, they make the decision	6.7	40.9
	Other household head is more knowledgeable or skilled to make the decision	86.7	45.5
	Other household head makes decisions about this activity/plot while I decide for other things	50.0	22.7
	Other (specify)	3.3	9.1
	*Observations*	30	22

The rationales women provided for claiming decision-making authority skewed heavily toward two explanations. First, women claimed authority on the basis of their labor contributions to plots, particularly in relation to planting date, fertilizer use, labor allocation, and variety use. Secondly, women cited their relative knowledge and skills relevant to the decisions, especially in relation to use of harvest. This indicates that labor contributions were a major driver of women’s involvement in operational decisions. For use of harvest and labor allocation decisions, women cited social norms that make these decisions their responsibility (43.6% and 10.8% respectively), although this reason was noticeably absent as a driver of women’s control over other decisions (Table 9).

Men who willingly ceded decision-making authority to their wives cited, first, that their spouse was more knowledgeable or skilled to make these decisions. Second, men alluded to the idea of “separate spheres,” noting that women made these decisions while men made others. Another common reason men ceded control over operational decisions was their wives’ labor contributions to the plot. Notably, men who ceded decision-making related to maize income to their wives (Table 10) were also unusually likely to cite conflict avoidance (50.0%).

### Rationales behind men’s control of decisions

Men’s reasons for controlling operational decisions skewed even more heavily than women’s toward their technical knowledge (Table 9). For men who reported making decisions about variety choice and fertilizer use, over 90% said that they did so due to their relative knowledge and skills on these subjects. Nearly 90% of men who control planting date decisions cited the same reason. Men were less likely than women to cite their labor contributions as reasons for controlling decisions, although this was generally the second most common reason men gave for exercising control. Critically, men who controlled decision-making, especially around inputs, often reported that financial control plays a role. Making decisions because they control the money was mentioned by 48.8% of men who control fertilizer decisions, 43.0% who control labor allocation decisions, 35.1% of men who control variety decisions, and 29.1% who control planting date decisions (Table 9). Focus group discussed underscored that men’s control over financial resources was an important factor shaping men and women’s involvement in decisions; women indicated they cannot control planting date decisions if they cannot or do not purchase seed themselves.

Among women who willingly ceded decision-making authority to their husbands, the top reason across decisions was the relative knowledge and skills of their spouse to make the decision. This mirrors the top reason that men ceded authority to their wives. Women who willingly left variety selection and fertilizer use decisions to their husband also linked these decision-making structures to control over financial resources (45.1% for variety selection, 45.4% for fertilizer use) and social norms around household headship (21.8% for variety selection, 16.0% for fertilizer use) at high rates relative to other management decisions (Table 10).

These data illustrate some agreement between spouses on the core rationales behind decision-making structures, but also many meaningful and statistically significant differences. Overall, men were more likely to stress that the reasons for their control over decisions were related to their knowledge and skills, while women were more likely to stress their labor contributions. There were several reasons given by women for decision-making arrangements that never or rarely were given by men. These included, notably, men’s control of financial resources, particularly for income use decisions, fertilizer use decisions, variety selection, labor allocation, and planting date. Gendered social norms that place decision-making power in men’s hands (or the head of household’s hands) also cropped up in relation to all decisions, but most notably income use and use of harvest. Land ownership was relatively less important in terms of shaping decision-making structures, although, unsurprisingly, it only emerged as an explanation for men’s control over decisions—never women’s.

## Discussion

### Prevalence of joint decision-making

These results have a number of implications for future research and programming related to agricultural development, training, and input delivery. One of the most relevant findings is the high prevalence of joint decision-making reported across a range of maize management decisions. These results likely reflect some response bias, given the prevalence of gender equality messages in development projects. However, the reported popularity of joint decision-making in maize plot management drastically complicates the common mandate to produce gender-disaggregated adoption data, which assumes relatively independent decision-making. Critical evaluation of gendered impact indicators is warranted, with increased attention to and accommodation of joint decision-making and joint use of technologies. Fundamentally, these findings are *not* an argument against gender-responsive technology design—rather, they underscore the importance of ensuring that the design and promotion of agricultural technologies and services account for the interests, priorities, and constraints of both men and women.

### Disagreement between spouses

A second critical finding relates to the level of disagreement between spouses concerning who makes decisions. This study adds to a growing body of literature documenting spousal disagreement and reasons for it (Acosta et al. 2020; Ambler et al. 2021; Anderson et al. 2017; Seymour and Peterman 2018; van Campenhout et al. 2022). There are no perfect ways to reconcile this disagreement; some studies opt to use only a single spouse’s response in analysis, chosen according to the relevance to study objectives. Other approaches average spouses’ responses to arrive at a compromise version of the “truth.” This study did not seek to reconcile responses, but instead built on Annan et al.’s (2020) framework for categorizing disagreement according to whether it constituted women “taking power” or men “giving power” to women. The results suggest women were more likely to claim power over input use decisions and more likely to be given power over decisions related to harvest uses–decisions that rely on women’s deep understanding of household needs.

This and other recent research on spousal disagreement has an important methodological implication: surveying a single spouse about the household only reflects one person’s perspective. This does not mean that every study must consult both spouses, necessarily, but does require that research designs account for the limitations of single-respondent approaches and evaluate which perspectives within the household are necessary to understand.

### Differences between strategic, operational, and financial decision-making

Because we evaluated decision-making across a range of activities, this study provided surprising insight into variation in decision-making structures for different management decisions. This included the high degree of joint decision-making reported for decisions typically considered to be male-dominated, such as those related to income derived from maize. Many existing datasets suggest that women have limited involvement in major household decisions, especially financial decisions. There are several possible explanations for the contrasting findings reported here, including a normative push to report joint decision-making, the relative jointness of maize production compared to other farm and household activities, and survey questions that were more specific than, for example, the Demographic and Health Survey’s (https://dhsprogram.com/), which is the basis for many assessments of women’s decision-making authority.

Of interest, however, is women’s lower rate of involvement in decisions that related to major financial expenditures that influence production potential (variety choice and fertilizer allocation). Women were granted greater control over decisions related to labor, which might also require financial expenditure, but reportedly relied on women’s specialized knowledge of who in the community is best suited to contribute to what type of farm labor.

The rationales provided for women’s vs. men’s control are telling in this context. While women were given slightly more control over operational decisions due to their knowledge, skills, and labor contributions, men were given slightly more control of fertilizer and variety decisions because they had more knowledge related to fertilizers and varieties and tended to control the financial resources used to purchase them (Tables 9 and 10). The fact that men evidently had more knowledge of fertilizer and varieties may be a product of men’s historic control of these decisions and more frequent interactions with agrodealers and extension. This points to the need for information campaigns and training that better target women. However, considering that many men involved women in decision-making primarily to avoid intrahousehold conflict, rather than because of their valuable knowledge, targeting training to women may be insufficient to strengthen their bargaining position. Gender norms, including perceptions of who in the household are the real “farmers,” remain a challenge.

### Conflating decision-making and women’s agency

This study provides support to recent research questioning the reliance on women’s involvement in decision-making as a proxy for their agency (Bernard et al. 2020; Seymour and Peterman 2018). While three different vignette options were provided in which the respondent was not the primary decision-maker, the most popular vignette among these, by far, was the “indifferent spouse” vignette that presented respondents as “opting out” of the decision. Both men and women very rarely identified with vignettes depicting exclusion, despite the fact that the language in the latter two vignettes was softened in pretesting to increase their appeal. Spouses appeared more likely to willingly delegate decision-making to the other (in line with the “separate spheres” model), which calls for more cautious consideration of core indicators of agency. However, with women most likely to opt out of decisions that directly impacted production potential (fertilizer use and variety choice), the prevailing separation of spheres may constrain women to decision-making spaces that do not allow for growth or empowerment.

### Limitations

The study has several limitations. First, the research questions and approach necessitated the exclusion of households without opposite-gender spousal co-heads who were physically present within the data collection window. The dynamics of decision-making in other types of households, including *de facto* woman-headed households with absent spouses, were beyond the scope of this study.

Second, although the use of vignettes to probe intrahousehold power dynamics helped ameliorate some sensitivities, this approach is not without weaknesses. Despite the careful tailoring of the vignettes for this study, the requirement that respondents identify with the “best aligned” scenario leads to a loss of some nuance. Additionally, using vignettes may have made it easier for respondents to identify with an “ideal” household regardless of the true degree of alignment with their circumstances. Survey-based approaches to evaluating decision-making, which often involve a series of relatively intrusive questions, may make it more difficult for respondents to hide information that cast the household in a negative light. However, such approaches place a greater burden on respondents and have potential to generate tension within respondent households. Additionally, the vignette approach does not appear to have mitigated men’s reluctance to admit to being excluded from decision-making by their spouse. Focus group discussions highlighted that men who fail to control their farm and household face stigma. Still, these discussions hinted that women may play an outsized role in decision-making in some households—one man cheekily commented that there were indeed “Lucys” in his village, in reference to the “marginalized spouse” vignette that depicted a man excluded from decision-making by his wife Lucy. However, almost no individual men selected vignettes depicting men’s exclusion during the surveys, likely indicating a reluctance to admit to these dynamics.

Finally, the study provides only a cross-sectional view of decision-making. It was clear from focus group discussions that negative outcomes in one season might lead to a change in decision-making structure the following season; if a man or woman made a decision independently that proved a mistake, the decision would more likely be made jointly the following year. Changes in household livelihood portfolios or cropping systems would likely also lead to shifts in decision-making authority. As such, intrahousehold decision-making dynamics should not be perceived as static.

## Conclusion

This study used vignettes to examine the intrahousehold dynamics of maize production decisions in Kenya. Our findings illustrate the prevalence of joint decision-making, underscoring the need to account for both men’s and women’s needs, priorities, and constraints in the design and delivery of agricultural technologies. The results also support shifting how we evaluate and understand farmer preferences and adoption decisions, with renewed focus on the (intra)household. This might mean shifting the focus from gender-disaggregated adoption to plot-level analysis and further exploring the importance of jointly managed farms to the success and wellbeing of women. These findings also call attention to longer term processes of negotiation between partners around household livelihoods, food security, and income (Doss and Quisumbing 2020), rather than a focus only on short-term individual decisions within households.

Although joint decision-making was the dominant model, close consideration of a range of maize management decisions showed that decision-making authority was slightly skewed for some decisions. Decisions around externally purchased inputs skewed toward men, which has implications for efforts at more inclusive marketing, outreach, and delivery. Management decisions that relied on knowledge of the household and community skewed toward women, including very crucial decisions around end-uses for harvested maize and operational decisions related to labor. However, the decisions women controlled had less direct impact on maize production potential, suggesting that within households, men still played a somewhat larger role in dictating the terms of production.

The reasons that spouses provided for decision-making arrangements, and particularly joint decision-making, offered important insights for gender research to further explore. While women were more likely to report that both spouses have important knowledge to contribute to a jointly made decision, men showed more concern for averting intrahousehold conflict. Men did not indicate as frequently that women’s knowledge is a key driver of joint decisions. The rationales that men and women provided for either claiming or delegating decision-making authority also suggest that social norms and unequal control over household resources continue to shape decision-making to a large extent, while women’s authority is derived from labor contributions. Ultimately, shifting decision-making dynamics to ensure women have a greater role in maize management will require transformation of gender relations. This includes building women farmers’ knowledge while increasing the visibility of their contributions to agriculture.

This study provides insight, too, into the need for improved methods around understanding plot management and jointness. First, the prevalence of household disagreement may necessitate speaking to multiple respondents within a household or strategically choosing whose perspective to collect. Additionally, evidence of variation in decision-making dynamics across maize management decisions complicates how we understand plot management. Surveys that ask respondents to identify plot managers may fail to capture the decision-making dynamics of greatest interest, particularly if “plot manager” is undefined or defined too generally. Future studies should aim for greater precision by focusing on the decisions or activities of greatest concern.

## Abbreviations

LSMS-ISA: World Bank’s Living Standards Measurement Study - Integrated Surveys on Agriculture
NGO: Non-governmental organization
FDC: Full disagreement between spouses, with both claiming authority
FDD: Full disagreement between spouses, with both delegating authority
MTP: Spousal disagreement scenario with man taking power
WTP: Spousal disagreement scenario with woman taking power
WGP: Spousal disagreement scenario with woman giving power
MGP: Spousal disagreement scenario with man giving power
